# Brain Microbial Populations in HIV/AIDS: α-Proteobacteria Predominate Independent of Host Immune Status

**DOI:** 10.1371/journal.pone.0054673

**Published:** 2013-01-23

**Authors:** William G. Branton, Kristofor K. Ellestad, Ferdinand Maingat, B. Matt Wheatley, Erling Rud, René L. Warren, Robert A. Holt, Michael G. Surette, Christopher Power

**Affiliations:** 1 Department of Medicine (Neurology), University of Alberta, Edmonton, Alberta, Canada; 2 Department of Medical Microbiology & Immunology, University of Alberta, Edmonton, Alberta, Canada; 3 Department of Surgery, University of Alberta, Edmonton, Alberta, Canada; 4 National Laboratory for HIV Pathogenesis, Health Canada, Ottawa, Ontario, Canada; 5 British Columbia Cancer Agency, Genome Sciences Centre, Vancouver, British Columbia, Canada; 6 Department of Medicine, McMaster University, Hamilton, Ontario, Canada; INSERM U1094, University of Limoges School of Medicine, France

## Abstract

The brain is assumed to be a sterile organ in the absence of disease although the impact of immune disruption is uncertain in terms of brain microbial diversity or quantity. To investigate microbial diversity and quantity in the brain, the profile of infectious agents was examined in pathologically normal and abnormal brains from persons with HIV/AIDS [HIV] (n = 12), other disease controls [ODC] (n = 14) and in cerebral surgical resections for epilepsy [SURG] (n = 6). Deep sequencing of cerebral white matter-derived RNA from the HIV (n = 4) and ODC (n = 4) patients and SURG (n = 2) groups revealed bacterially-encoded 16 s RNA sequences in all brain specimens with α-proteobacteria representing over 70% of bacterial sequences while the other 30% of bacterial classes varied widely. Bacterial rRNA was detected in white matter glial cells by *in situ* hybridization and peptidoglycan immunoreactivity was also localized principally in glia in human brains. Analyses of amplified bacterial 16 s rRNA sequences disclosed that Proteobacteria was the principal bacterial phylum in all human brain samples with similar bacterial rRNA quantities in HIV and ODC groups despite increased host neuroimmune responses in the HIV group. Exogenous viruses including bacteriophage and human herpes viruses-4, -5 and -6 were detected variably in autopsied brains from both clinical groups. Brains from SIV- and SHIV-infected macaques displayed a profile of bacterial phyla also dominated by Proteobacteria but bacterial sequences were not detected in experimentally FIV-infected cat or *RAG1^−/−^* mouse brains. Intracerebral implantation of human brain homogenates into *RAG1^−/−^* mice revealed a preponderance of α-proteobacteria 16 s RNA sequences in the brains of recipient mice at 7 weeks post-implantation, which was abrogated by prior heat-treatment of the brain homogenate. Thus, α-proteobacteria represented the major bacterial component of the primate brain’s microbiome regardless of underlying immune status, which could be transferred into naïve hosts leading to microbial persistence in the brain.

## Introduction

The existence of commensal microbes that colonize organs within the human body has long been recognized and termed the microbiome [1], [2]. Once thought of as harmless tenants, it has become increasingly obvious that the microbiome is not comprised of passive passengers but of specific infectious agents that participate in shaping the metabolic and immune status of the host [3], [4]. The composition of the human microbiome at several body sites including skin, oral cavity, gastrointestinal tract, nasopharyngeal passage and urogenital tract are subjects of intensive study [2], [5], [6], [7], revealing both local effects at these sites but also systemic effects, including influencing inflammatory or metabolic functions in privileged organs such as the brain [8], [9], [10]. These latter studies have reported complex bacterial populations with wide variations in individual bacterial constituents at the species level, both in serially sampled humans and across groups of humans. The composition of the microbiome at the phylum level is consistent throughout the body with the exception of the skin, where diverse population structures occur at different sites [11], [12]. In most body sites sampled to date, Firmicutes and Bacteriodetes represent the dominant phyla with substantial numbers of Actinobacteria and Proteobacteria detected, while other phyla comprise a small proportion of the remaining species. Importantly, body sites previously presumed to be sterile in healthy humans, such as the vascular endothelium, have been shown to be colonized without apparent signs of disease. In contrast, diseases such as biliary cirrhosis [13], [14], atherosclerosis [15] and aortic aneurysms [16], previously considered attributable to autoimmunity or pathogenic metabolites, are now hypothesized to be induced or exacerbated by infectious agents, possibly as part of local microbiomes

Human immunodeficiency virus type 1 (HIV-1) infection with the ensuing development of the acquired immunodeficiency syndrome (AIDS) is a recognized cause of perturbation within microbiomes within the gut and other organs [17]. The altered immune status of HIV/AIDS patients is associated with qualitative and quantitative shifts in microbiome diversity and quantity as well as increasing the likelihood of microbial translocation from the gut to other organs [18]. These events occur because of reduced immune surveillance and damaged barrier function at epithelial and endothelial sites, including damage to the blood-brain barrier [19]. Multiple studies have shown the blood-brain barrier to be more permeable in HIV/AIDS, implying this circumstance might contribute to HIV-associated neurological disease [20], [21]. Given the emerging appreciation of microbiome disruption together with the damage to the brain injury during HIV/AIDS, we hypothesized that brain tissue from patients with HIV/AIDS might contain greater microbial quantities and perhaps diversity, derived largely from other organs such as the gut, which has a well-established microbiome and altered function during HIV infection. In fact, the present studies showed that all examined human brains contained bacterially-expressed RNA and associated products together with a predominance of α-proteobacteria, regardless of the patients’ immune status and concurrent diseases. Moreover, a similar bacterial population was observed in the brains of non-human primates. This microbial population found in the human brain could be transmitted into immunodeficient mice and subsequently recovered 7 weeks later suggesting the brain was tolerant to the presence of certain bacterial species

## Results

### Bacterial RNA Detection in Brain Tissue by Deep Sequencing

Massively parallel sequencing without prior amplification was performed using cDNA derived from total RNA that was extracted from autopsy-derived cerebral white matter of HIV/AIDS (HIV) (n = 4: HIV1-4) and other disease control (ODC) (n = 4: ODC1-4) patients, as well as from white matter extracted from brain tissue collected during surgical (SURG) resections for epilepsy (n = 2: SURG 1–2) (Table 1). The sequence tag lengths varied between 36 and 77 nucleotides and the maximum number of unambiguously identified tags ranged from 5,500,000 to 11,700,000 depending on the individual patient sample. Of these 3,000,000–7,000,000 tags per patient mapped to the human genome (∼20,000 open reading frames); 200 to 21,000 of the remaining tags mapped unambiguously to bacterial or viral sequences. Following contaminant screening based on non-human libraries, all sequence tags were shown to be intrinsic to the brain-derived libraries.

**Table 1:** Clinical features, sex and neuropathological diagnoses.

Group	Mean Age^1^	Sex	Neuropathology^5^ [Patient identity number]
ODC^2^	62±11.6	(Male n = 8)	Cerebral infarct [ODC1, 8, 11]
(n = 14)			Acute meningitis with abscess [ODC3]
			Rabies encephalitis [ODC4]
			Toxoplasmic encephalitis [ODC5]
			Hypoxic Ischemic encephalopathy [ODC6]
			Sepsis with hypoxic ischemic encephalopathy [ODC12]
			Multiple sclerosis [ODC13]
			Rasmussen’s encephalitis [ODC14]
			Normal brain (endocarditis; drug overdose; leukemia etc) [ODC2, 7, 9, 10]
HIV^3^	38±5.9	(Male n = 7)	HIV encephalitis [HIV1, 7, 9]
(n = 12)			Toxoplasmic encephalitis [HIV2]
			CMV/HSV encephalitis [HIV3, 8]
			Microglial nodule encephalitis [HIV4, 6, 11]
			Multifocal necrotizing leukoencephalopathy [HIV5]
			Hypoxic Ischemic encephalopathy [HIV10]
			Normal brain [HIV12]
SURG^4^	24±20	Male (n = 5)	Normal brain, proximal to epileptic focus
(n = 6)			

1 Mean ± standard deviation.

2 ODC – Other Disease Controls.

3 HIV – All HIV-infected patients were AIDS-defined.

4 SURG –Surgical resection for an epileptogenic lesion.

5 All molecular studies were performed using cerebral white matter.

Analysis of the present dataset revealed sequence tags mapping unambiguously to 173 different bacterial- and phage- -derived sequences. Several bacterial classes were represented as high tag numbers in multiple patient samples compared to the host gene, *βIII-tubulin*, which was expressed in all samples (Table 2). α-proteobacteria sequence tags were identified in all patient samples and showed the greatest similarity to members of the Sphingomonadaceae family, which were of particular interest because of the ability of several members of this family to synthesize and incorporate sphingolipids including ceramide into their outer membranes [22]. The most abundant bacterial tags matched most closely with the *Sphingomonas wittichii* RW1 genome and the majority of which were found to map to sequences within the 23 s rRNA loci. However, the matches to bacterial sequences had restricted discriminating power because they were based on short sequence tags and were considered to be matches limited only to the class level.

**Table 2:** Total sequence tag counts per patient for principal bacterial classes detected in brain^1^.

	Other Disease Controls				HIV/AIDS				Surgical Samples	
Bacterial Class	ODC1	ODC2	ODC3	ODC4	HIV1	HIV2	HIV3	HIV4	SURG1	SURG2
α-Proteobacterium	168	39	98	27	41	49	37	17	3	19
Actinobacterium	13	8	6	7	17	9	24	4	0	0
β-Proteobacterium	5	3	0	0	0	0	1	0	0	0
γ-Proteobacterium	2	0	2	2	0	4	1	0	0	0
βIII*-tubulin* 2	35	291	36	20	87	223	65	73	47	582

1 All sequence tag counts were normalized across experiments.

2 Host gene.

**Figure 1:**
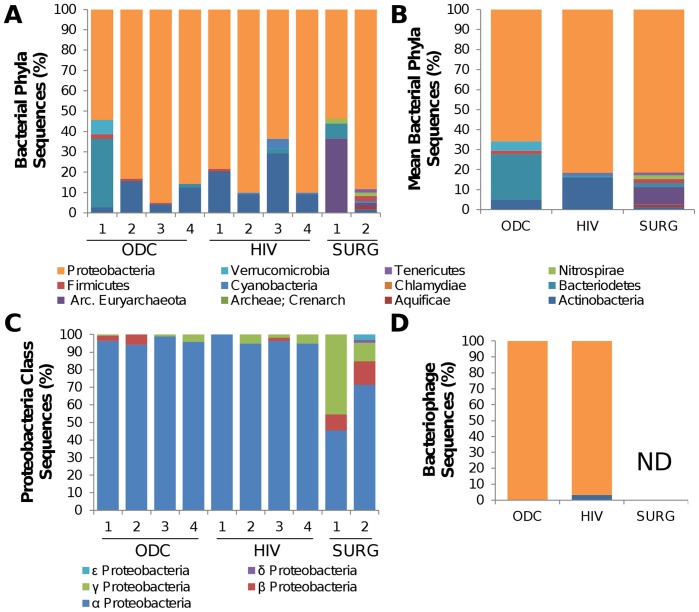
Deep sequencing detection of bacterial and bacteriophage RNA sequences in human brain. (**A**) Total sequence tags that were unambiguously identified as belonging to a bacterial phylum were grouped for each patient from which the percentages for each phylum were displayed. All patients showed a predominance of Proteobacteria-associated sequences. (B) Despite inter-individual variability the mean percentage of Proteobacteria sequences among the HIV, ODC and SURG groups was similar. (C) The majority of bacterial sequences identified in all patient samples belonged to the Proteobacteria phylum, which showed the greatest similarity to the α-proteobacteria class. (D) The majority of bacteriophage sequences identified matched Proteobacteria-tropic phage sequences although bacteriophage sequences were not detected in the SURG samples.

The relative abundance of several bacterial phyla in the present brain samples was determined using normalized tag counts from each sample (Figure 1). The most abundantly detected phylum in all samples was Proteobacteria, based on a patient-by-patient analysis (Figure 1A). Moreover, one of the dominant phyla in other organs (Firmicutes) was undetectable in most brain-derived RNA samples by this approach. The next most abundant phylum in most samples was Actinobacteria. The identities of minor bacterial constituents showed variation between different patients. Surgically-derived cerebral samples (SURG-1 and SURG-2) showed a similar predominance of Proteobacteria, but in contrast to the autopsy-derived specimens, Actinobacteria were not among the principal constituents (Figure 1B). The sequences mapping to members of the Proteobacteria phylum were examined at the class level, revealing that α-proteobacteria was the most frequently detected class of Proteobacteria in all brain samples (Figure 1C). In agreement with the distribution of bacteria in the present samples, the majority of bacteriophage sequences detected in autopsy-derived cerebral white matter specimens corresponded to Proteobacteria-tropic phage RNA (Figure 1D). Tags mapping to bacteriophage sequences were not identified in the surgically-derived samples. HIV-1 sequence-specific tags were also detected in brain specimens from the HIV group but other viral RNA sequences were infrequently detected by deep sequencing in the present specimens with the exception of the specimen from the ODC patient (ODC4) diagnosed with rabies encephalitis, from which in excess of 20,000 rabies virus sequence tags, spanning the entire viral genome, were identified [23]. To explore abundance of viruses known to infect the brain in more detail, the presence of viral genomes was investigated in total RNA and genomic DNA from HIV and ODC white matter samples. Cytomegalovirus (HHV-5), HHV-6A/B and Epstein-Barr virus (HHV-4) sequences were variably present in specimens from both groups of autopsied samples but other DNA and RNA viruses were infrequently detected in HIV or ODC brain specimens (Table 3).

**Table 3:** Detection of viral genomes in brain-derived cDNA or gDNA.^1^

Patient group	HHV-6 A/B	VZV	EBV	HSV-1	CMV	KSHV	SAFV^2^	CoV^2^
HIV1-6	1/6	0/6	3/6	0/6	4/6	1/9	0/6	1/6
ODC1-8	3/8	0/8	1/8	0/9	3/8	0/7	0/6	1/6

1 Viral detection based on PCR or RT-PCR analyses.

2 Dectection of virus-encoded RNA by RT-PCR.

### Host Responses in the Human Brain

**Figure 2:**
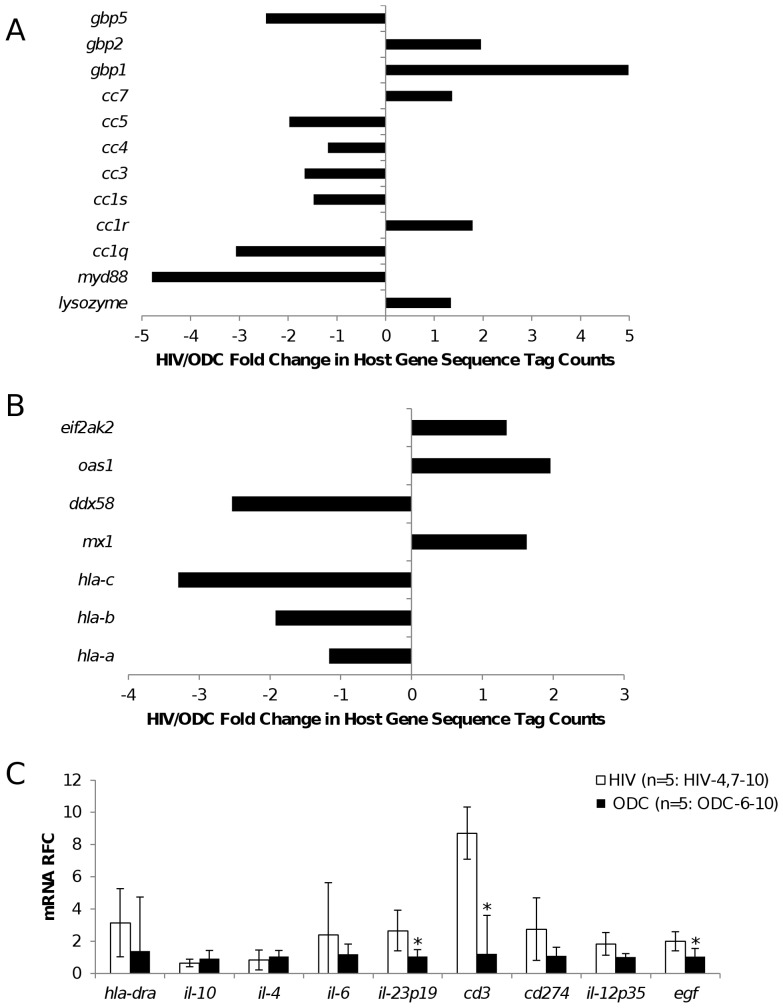
Human brain host gene responses. Analysis of human transcript sequences implicated in (A) bacterial and (B) viral host responses identified by deep sequencing of brain-derived RNA showed variability within clinical groups with many genes showing a relative reduction or induction in expression in the HIV relative to ODC brain specimens. (C) Host neuroimmune responses were assessed as transcript abundance in terms of relative fold change (RFC) by real time RT-PCR, showing a general increase in pro-inflammatory gene expression among the HIV (n = 5) compared to ODC (n = 5) brain specimens with significant changes in *cd3ε, IL-12* and *egf* expression.

As both bacterial and viral genomes were detected in the present brain specimens, we examined host genes known to participate in responses to infections. Several genes exhibited variable expression in HIV relative to ODC brains based on tag counts from the whole transcriptome analysis with *myd88* showing marked suppression (4-fold) in HIV-derived brain specimens compared to the ODC group; conversely, *lysozyme* was induced in the HIV group (Figure 2A). Analysis of host genes germane to viral infections disclosed that antiviral genes such as *mx1* and *oas1* were induced in the HIV brains while MHC Class I genes (*hla-c, -b* and *-a*) were relatively suppressed in the same group (Figure 2B). Further analysis by real time RT-PCR showed that transcripts encoded by multiple genes implicated in host responses to viral infections of the brain were increased in the HIV/AIDS brains (HIV4, 7–10, n = 5)) with *cd3ε, egf* and *il-23* showing significant increases compared to ODC brains (ODC6-10, n = 5)) (Figure 2C). Network analyses showed associations between bacterial sequence tag quantities and variable expression of host genes implicated in essential cellular structure and maintenance functions (Figure S1). From these studies, it was evident that there was a differential expression of host neuroimmune genes in the clinical groups, emphasizing the divergent biological environments in the HIV compared to the ODC brain specimens.

### Immunodetection and In Situ Hybridization of Bacterial Products and Genomes

**Figure 3:**
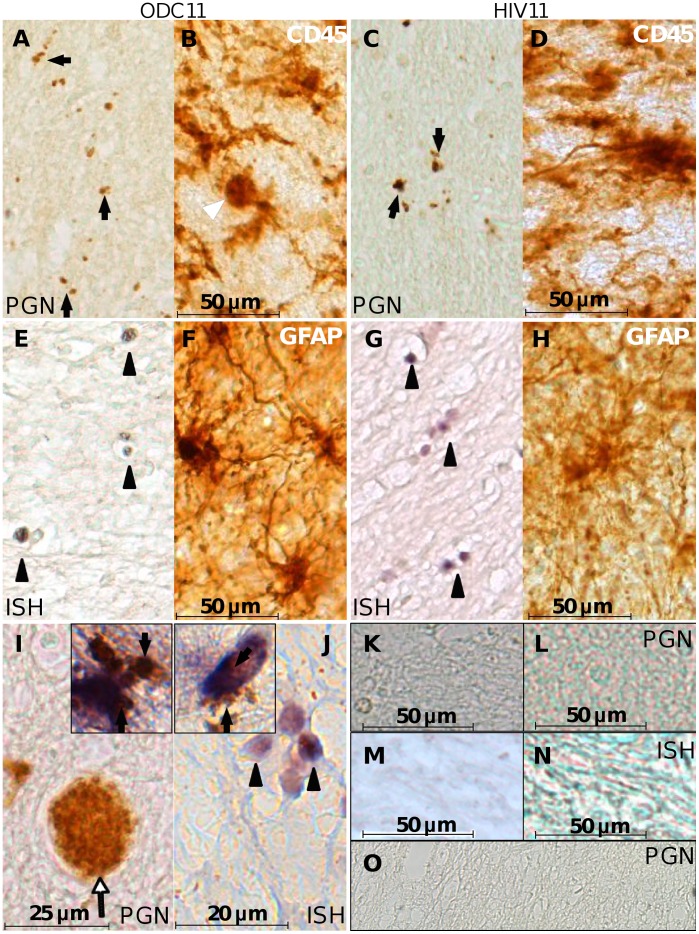
Bacterial detection in human brain. Autopsy-derived ODC (A), and HIV (C) brain specimens were immunolabeled with anti-peptidoglycan antibody. Peptidoglycan (PGN)-positive bodies (arrows) were morphologically consistent with bacteria and smaller than CD45 immunopositive microglia from ODC (B) and HIV (D) patients, imaged at the same magnification. Double DIG-labeled EUB 338 probe *in situ* hybridization (ISH) against the 16 s rRNA gene was hybridized with slides from the same ODC (E) and HIV (G) patients and labeled with alkaline phosphatase-conjugated sheep anti-DIG FAB` fragments and stained with NBT/BCIP. ISH-positive bodies featured morphology resembling bacteria (arrow heads) and were smaller than GFAP immunopositive astrocytes in ODC (F) and HIV (H) patient sections. Peptidoglycan-labeled cells with both spherical and rod morphology were observed within the brain parenchyma and in a blood vessel (I) (White arrow) of an ODC patient. Peptidoglycan immunopositive bodies were observed within the cytoplasm of GFAP-immunolabeled astrocytes (I, inset) and Iba-1 immunolabeled microglia (J, inset). Spherical clusters of EUB 338 hybridized cells J) were evident (black arrowheads). Slides from the same ODC11 (K) and HIV11 (L) patients were processed under identical conditions except that the primary antibody was omitted. A scrambled DIG-labeled probe was hybridized to slides from the ODC11 (M) and HIV11 (N) under identical conditions used for the EUB 338 probe. In all cases specific signals were not detected. (Original magnification 200×). (O) A section from the forebrain of one of the FIV-infected cats was immunostained with the anti-PGN antibody and developed with DAB with no detectable signal. (Bar: A–H, 50 microns; I, 25 microns; J, 20 microns; K–N, 50 microns) (Magnification: A–H, 200×; I, 400×; J, 600×; I and J insets, 600×).

The above molecular findings prompted investigation of bacterial antigens and genetic material within the cerebral white matter of HIV and ODC persons. Immunoreactivity to the ubiquitous bacterial wall constituent, peptidoglycan (PGN), was observed in white matter of both ODC (Figure 3A, arrow) and HIV (Figure 3C, arrow) patients as particulate immunostaining, albeit less abundant than CD45-immunopostive microglia in both groups (Figure 3B and D; arrowhead indicating microglial cell body). Peptidoglycan immunoreactivity was also evident within blood vessels (Figure 3I, arrow) and was co-localized with immunoreactivity for GFAP (astrocytes) (Figure 3I, inset) and Iba-1 (microglia and macrophages) (Figure 3J, inset). Other HIV patients with or without encephalitis showed similar patterns of peptidoglycan staining to that observed among ODC patients (Figure S2). Control slides in which the primary antibody was omitted did not show immunoreactivity (Figure 3K [ODC] and 3L [HIV]), nor did cerebral sections from FIV-infected cats reared in a specific pathogen free facility (Figure 3O).

*In situ* hybridization (ISH) performed on brain sections, using a probe hybridizing to a conserved sequence within the bacterial 16 s rRNA encoding gene, also revealed positive detection of spherical structures, 2–6 µm in size, resembling bacteria and clusters thereof in ODC (Figure 3E, arrowhead) and HIV (Figure 3G arrowhead) patients’ brains but again fewer and smaller than GFAP-immunopositive fibrous astrocytes in both clinical groups (Figure 3F and H). As observed above with peptidoglycan staining, ISH-positive structures were found within cells and in the extracellular matrix at high magnification (Figure 3J, arrowhead). Sections from the same patients hybridized with a probe encoding a scrambled sequence did not show specific labeling (Figure 3M (ODC) and 3N (HIV)).

### Amplification and Sequencing of Brain-derived Bacterial 16 s rRNA

**Figure 4:**
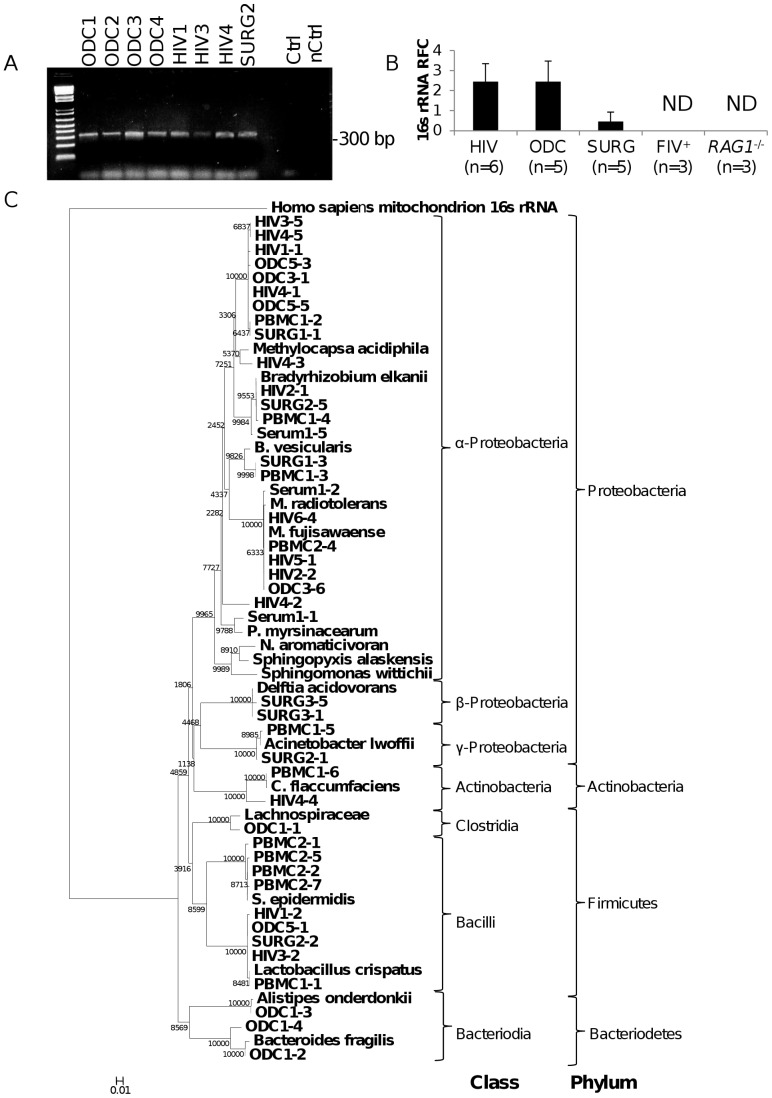
Phylogenetic analyses of bacterial 16 s rRNA sequences from human brain and blood. (A) Representative ethidium bromide gels show the amplification of a single band by a nested PCR protocol but in matched (nested) water controls, a product was not observed. (B) Real time RT-PCR showed similar levels of mean 16 s rRNA amplicon quantities in HIV and ODC brain samples, however SURG samples showed lower amplicon levels of 16 s rRNA products. (C) Phylogenetic analyses were determined among cloned amplicons generated from brain cDNA from ODC (n = 6), HIV (n = 6), SURG (n = 3) as well as cDNA from PBMCs (n = 2) and serum (n = 2) from healthy volunteers (PBMC 1,2; Serum 1) using the universal bacterial 16 s primers 514F and 806R and the equivalent region of various published 16 s rRNA genes. The alignments were used to generate a neighbour joining tree using 10,000 bootstrap trials. There was a predominance of α-proteobacteria in brain-derived 16 s rRNA clones although other classes, e.g., β- and γ-proteobacteria, and Bacilli, were amplified from blood-derived cDNA.

To confirm the above deep sequencing detection of bacterial RNA, real time RT-PCR was performed amplifying distinct regions of bacterial 16 s ribosomal RNA (rRNA) in cDNA generated from autopsy-derived cerebral white matter of HIV (n = 6) and ODC (n = 6) patients, as well as cerebral white matter from surgical specimens (SURG (n = 6)). PCR conditions using several primer pairs were optimized in advance by colony PCRs using *E. coli* DH5α as a template. All PCR reagents were confirmed to be free of contaminants using ultrapure water as no-template controls for all primer pairs (Figure S3A). In all brain samples examined, bacterial 16 s rRNA encoding sequences were detected, although PCR products were not detected in reaction in which there was no template (Figure 4A). The mean level of 16 s rRNA-encoding amplicons was similar in the HIV and ODC autopsied brain samples (Figure 4B) but greater than in the SURG specimens; 16 s rRNA sequences were not detected in the brains of FIV-infected cats (n = 3) and *RAG1^−/−^* mice (n = 3) (Figure 4B). Of note, amplification of bacterially-encoded *groEL* transcripts generated amplicons that were consistently detected at high cycle thresholds of ≥34–35, suggesting that bacterial transcript levels were low in human brains. To control for contaminated reagents and equipment used throughout the tissue collection and RT-PCR process cDNA synthesis was performed using ultrapure water and ultrapure water used to rinse a surgical tool (hemostat) opened in the operating theatre during the collection of SURG6 as the substrate in parallel with a subset of brain-derived RNA using the same reagents and equipment. This control product, used as a PCR template, failed to amplify an amplicon corresponding to 16 s rRNA or any other amplicons (Figure S3B). These studies verified the presence of bacterial RNA in human brain samples but also validated the methodologies used herein by showing all of the negative controls did not yield PCR products.

Based on amplification and cloning of at least 5 clones per patient, a neighbor-joining phylogenetic tree was constructed predicated on representative clones exhibiting consensus sequences as well as unique clones with the nearest bacterial 16 s rRNA matches identified by BLAST analysis, which was rooted to human mitochondrial 16 s rRNA (Figure 4C). For comparison, the same analysis was conducted using cDNA derived from serum and peripheral blood mononuclear cells (PBMCs) from healthy donors (n = 2). 16 s rRNA V3–V4 sequences from HIV and ODC patients overlapped phylogenetically and with the cloned sequences derived from SURG specimens. Most sequences derived from blood corresponded to the Bacilli class and diverged from brain-derived sequences (Figure 4C). Proteobacteria sequences predominated among the brain-derived clones. When interpreted at the class level, the predominant class was α-proteobacteria, with some γ- and δ-proteobacteria representation. Of note, the sequences amplified from ODC patient 1 (ODC1) were the only sequences belonging to the Bacteriodetes phylum. The patient’s history of hemorrhagic cerebral infarction, together with the distinct profile of 16 s RNA sequences observed in this patient might represent disruption of the blood-brain barrier, resulting in infiltration of atypical organisms that differed from the other brain specimens. Amplification of the 16 s rRNA V8 sequences again showed similar amplicon levels among ODC and HIV brain specimens (Figure S4A). Amplified V8 sequences from brain were identified as belonging to different classes of bacteria belonging to α-, β-, γ- and δ-proteobacteria, and Bacilli although β-protobacteria was the predominant class (Figure S4B). Thus, these studies validated the identities of the bacterial classes detected in human brains by deep sequencing.

**Figure 5:**
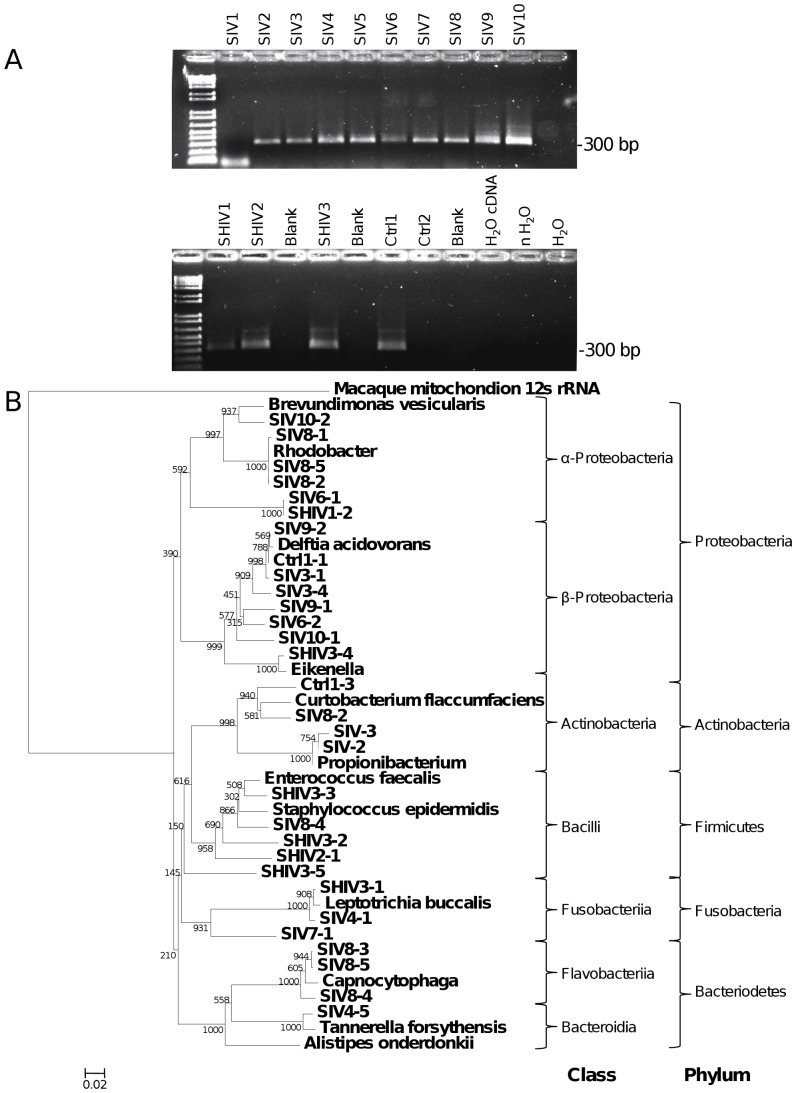
16 s rRNA sequences from brain-derived cDNA of cynomolgus macaques. (A) Ethidium bromide-stained agarose gel showed the amplicon generated by primers 16 s514F and 16 s 806R from SIV-infected macaques 1–10, SHIV-infected macaques 1–3 and FIV-challenged macaque 1 and 2. cDNA synthesized with ultrapure water, water carried through both rounds of nested PCR and water used as template only in the final round of PCR were all included as negative controls. (B) Phylogenetic analysis of 16 s rRNA region sequences amplified by the primers 514F and 806R derived from macaque brain specimens. Clustal alignments were generated comparing amplicon sequences with the equivalent position of published 16 s rRNA sequences identified by BLAST analysis. The Neighbor joining tree was generated based on 10,000 bootstrap trials and rooted on the macaque mitochondrial 12 s rRNA sequence. Again, these data showed a predominance of Proteobacteria in brain-derived 16 s rRNA clones although other bacteria, e.g., Actinobacter and Bacilli, were detected.

Since all of the human brain specimens examined herein exhibited bacterial sequences while non-primate experimental animals showed no evidence of bacterial infection despite being immunosuppressed and brains were collected with similar protocols for handling and preparing tissues, bacterial RNA sequences were investigated among experimental non-human primates. In all but one of the cynomolgus macaques’ brains infected with SIV (n = 10) and all animals infected with SHIV (n = 3) 16 s rRNA V3–V4 sequences were observed, (Figure 5A) but of the FIV-challenged macaques (n = 2), the brain of one animal (Control 2) did not exhibit detectable bacterial sequences; this latter animal was housed in a SPF facility. Phylogenetic analysis of 16 s rRNA sequences derived from macaques showed more diversity than among the human brains but again displayed sequences largely matching Proteobacteria (Figure 5B). These studies of non-human primates recapitulated the above observations in human brains derived from both deep sequencing and amplification followed by cloning and conventional sequencing.

### Cerebral Bacterial Transmission

**Figure 6:**
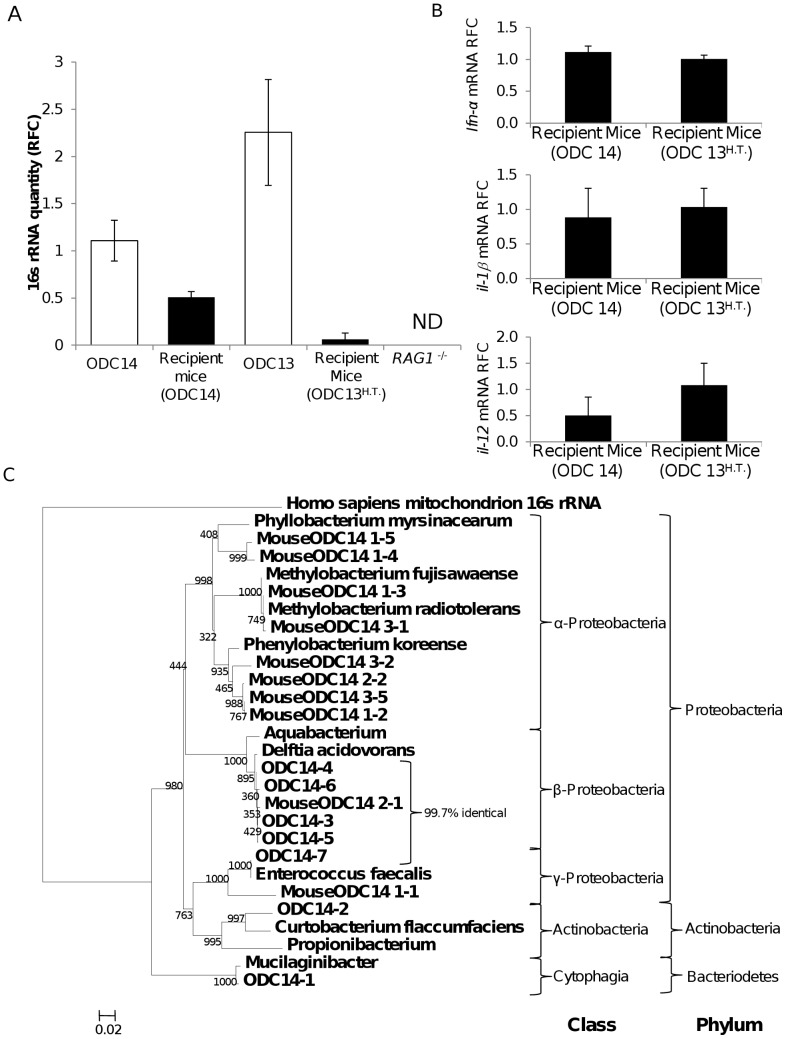
Transmission of bacteria from human brain to *RAG1^−/−^* mice brains. (A) Comparison of the relative quantity of 16 s rRNA in human and matched recipient mouse brains showed that mean bacterial rRNA levels in recipient mice were present at ∼40% of the mean ODC14 brain homogenate rRNA levels but less than 1% for the heated-treated ODC13 brain homogenate (prior to heat-treatment), measured by real time RT-PCR. (B) Mouse host inflammatory gene transcripts (*ifn-α, il-1β* and *il-12*) in mouse brains were not induced by implantation of untreated compared to heat-treated human brain homogenates. (C) The bacterial 16 s rRNA sequences from the ODC14 brain homogenates and the recipient mouse ODC14 brains were similar with substantial phylogenetic overlap between the bacterial genera, as evidenced by the 97% similarity for *Delftia acidovorans* sequences.

As bacteria were evident in brains of persons with and without apparent neurological disease (Figure 1A) and bacterial sequences were not present in *RAG1^−/−^* mice (Figure 4B), we investigated the transmissibility of brain-derived bacteria in these animals because of their apparent sterile brains and their minimal adaptive immune responses, which might potentiate any disease mechanisms. Homogenized human brain samples (ODC13 and 14) with and without prior heat treatment, respectively, of the homogenate were stereotactically implanted into the striatum of *RAG1^−/−^* mice. The implanted mice were maintained for seven weeks and then sacrificed and total RNA was extracted from the brains. In mice that received unheated (ODC14) brain homogenates, 16 s RNA V3–V4 sequences were detected at levels that were at 40% abundance of the original implanted human brain homogenate (Figure 6A). Conversely, 2 of the three mice that received heat-treated brain homogenates (ODC13^H.T.^) did not contain detectable 16 s rRNA while the third animal contained 16 s rRNA at levels >1% of the original implanted human (ODC13) tissue. The expression of *ifn-α, il-1β and il-12* (mouse) transcripts was examined in the brains of the both groups of recipient mice; these analyses revealed implantation of untreated brain homogenates did not evoke increased or sustained neuroimmune responses compared to animals’ brains that received the heat-treated brain homogenates (Figure 6B). Expression of essential host gene transcripts such as calreticulin was also similar in both groups and neurological deficits were not evident in either experimental group of mice. Sequencing of 16 s rRNA from the initial untreated human brain homogenate (ODC14) and the corresponding recipient mouse brains showed closely related sequences with phylogenic clustering, again pointing to a high proportion of Proteobacteria-derived sequences (Figure 6C). Taken together, these results indicated that human brain-associated bacteria could be transferred into and recovered from the brains of recipient mice at comparable bacterial RNA quantities and diversity to the original implanted human brain homogenate.

## Discussion

The present study reports the first deep sequencing analysis of microbial populations within the normal appearing tissue in the human brain with confirmatory methodologies that highlighted the consistent presence of bacterial ribosomal RNA and associated bacterial products. The majority of the bacterial RNA sequences identified in all human and nonhuman primate brains were encoded by members of the α-proteobacteria class, regardless of the underlying disease process. This restricted bacterial diversity observed in normal brain tissues is in contrast to the findings of a wide variety of organisms from other groups studying brain abscesses [24], [25]; many of which display colonization by bacteria from other tissues. This dichotomy in findings suggests that the bacteria identified in normal appearing tissue in the present studies might be are outcompeted and/or supplanted by organisms from other body sites in the case of intracerebral abscesses. Peptidoglycan immunoreactivity and *in situ* hybridization detection of bacterial rRNA were apparent within glial cells and in the extracellular space. *In vivo* cerebral implantation of human brain homogenates into mice showed that the 16 s RNA sequences recovered from recipient animals’ brains were conserved and expressed at levels similar to the initial human brain homogenate. Transmission was interrupted by heat treatment of the initial homogenate, implying that viable bacteria were required for transmission. These findings indicated that bacteria were present in the primate brain and do not appear to be derived from the predominant populations at other human body sites. Indeed, the predominance of α-proteobacteria in the brain is unparalleled in other body sites where the microbiomes are dominated by Firmicutes, Bacteriodetes and Actinobacteria.

The bacterial rRNA V3–V4 region-based detection of α-proteobacteria in the present studies was supported by the unbiased (non-amplified) deep sequencing. The relatively greater abundance of β-proteobacteria identified using the V8 region amplicon might reflect a bias because of the poor discriminating power of the smaller V8 amplicon or the restricted diversity within this region relative to the analyzed V3–V4 region amplicon, leading to imprecise assignment of sequences to this closely related class. There might also be a bias introduced by the PCR conditions, despite the use of universal primers, resulting in an over-representation of β-proteobacteria sequences. This finding needs to be clarified through culturing of brain specimens as part of future analyses to identify definitively the components of the primate brain’s microbiome at the species level. Of interest, α-proteobacteria comprise one of the most diverse bacterial classes with wide spread biological niches and actions including detoxifying effects in the environment [22], [26]. The acquisition of this class of infectious agents by the brain might represent a beneficial organ-specific adaptation.

The potential for contamination of samples at any stage of the tissue preparation, together with blood contamination was considered throughout the present studies. Controls for all lots of reagents were used throughout this work at all steps of the RNA extraction, cDNA synthesis and conventional and quantitative RT-PCR. The current tissues varied in harvest times and sources; for example, autopsy times ranged from 12–24 hr, while surgical samples were collected under sterile conditions and immediately frozen on dry ice in the operating room. To confirm that 16 s rRNA amplicons identified were intrinsic to the tissue and not introduced during sample preparation RNA extraction, cDNA synthesis and PCR, extensive reagent and equipment controls were used throughout these studies up to and including having the entire process repeated by different personnel in another facility using different lots of consumables and reagents (Figure S3B). White matter was intentionally selected to limit blood contamination of samples; additionally, bacterial genome and products were detected in brain parenchyma remote from blood vessels and within cells (glia) known to phagocytose foreign materials. Likewise, the current simian brain samples were harvested and processed by different investigators in a distant facility, yet the same phyla were predominant in the majority of macaque brains examined. Brain samples from experimental animals reared in SPF conditions with concurrent immunosuppression (*RAG1^−/−^* mice and FIV-infected cats) were included as controls within these studies but did not show bacterial 16 s rRNA sequences in brain despite being processed in an identical manner to the human and macaque brains, even with prior amplification steps and identical preparatory methods. Despite the diversity of techniques and sources of the present brain tissues, α-proteobacteria represented the most prevalent bacterial class discovered within the human brain, which was in contrast to blood samples. The consistency of α-proteobacteria detection in primate brains but not in SPF experimental animal brains emphasized the reliability and specificity of the present observations.

Several of the bacterial classes observed in the present studies have been associated previously with human diseases. For example, an organism similar to many of the 16 s V8 region sequences, *Delftia acidovorans* has been implicated in endocarditis [27], bacteremia [28], [29], corneal keratitis [30] and urinary tract infections [31]. In addition to causing infections in compromised patients, *D. acidovorans* and other members of the Comamonadaceae have been identified as part of the bacterial community in the arterial wall in patients who have had aortic aneurysms [16]. *D.* *acidovorans* has also been isolated from cerebrospinal fluid, sputum, urine, pharynx and wounds without concurrent signs of disease [32]. Taken together, this ubiquitous environmental organism likely represents a commensal organism with wide tissue distribution that can act as an opportunistic pathogen in vulnerable patients. As such, the prevalence of sequences showing similarity to *Delftia sp.* in the majority of patients in all the present clinical groups bears close scrutiny as host specific factors might determine whether or not this organism contributes to brain disease. Two HIV/AIDS patients exhibited sequences similar to Alcaligenaceae; members of this family have been identified as components of the normal flora in Peyer’s patches [33], but members have also been implicated in endocarditis [34], bacteremia [35], [36] and meningitis with or without ventriculitis in neonates [37], [38], HIV-infected persons [39], other immunocompromised adults [40] or following invasive surgery [41]. Thus, identifying members of this family may represent translocation of commensal gut organisms to the brain or a previously unidentified subclinical infection in these patients.

As the surgical cerebral resections were collected from patients as part of a procedure for epilepsy, they represent the samples with the lowest probability of *ex vivo* changes, such as RNA degradation or a possible artifact of bacterial growth. Differences in the bacterial populations between the surgically- and autopsy-derived specimens could represent *post mortem*–related differential levels of RNA degradation or changes in microbial replication. The >5 fold increase in the representation of Archea in the cerebral surgical specimens relative to the ODC group was interesting, as members of this kingdom were undetectable in all but one autopsy brain specimen.

In an organ widely assumed to be free of infectious agents in the absence of a specific disease process, autopsied and surgically-derived human brain specimens showed a restricted but distinct bacterial population in the present studies, which was composed of bacterial classes chiefly recognized in the physical environment, i.e., soil and water. The sources of these agents might include oral consumption or inhalation with eventual transport to the brain as intracellular agents in activated leukocytes trafficking into the brain. The brain is constantly surveyed by trafficking leukocytes (activated lymphocytes and macrophages), which provide a Trojan horse mechanism for microbial entry into the nervous system across the blood brain barrier. In fact, this mechanism is well recognized as a route by which viruses infect the brain and likely underlies the detection of herpes viruses in both the HIV and ODC brains. Corroborating this latter point is the report of peptidoglycan detection in brain lesions from multiple sclerosis patients, which are heavily infiltrated with blood-derived leukocytes [42]. Since bacteria express multiple molecules that activate immune signaling cascades by engaging Toll- or NOD-like receptors, etc., their capacity for influencing brain function [43] is immense. Hence, studies focused on delineating the brain’s microbiome at the species level together with their individual effects on host cell physiology might lead to a greater understanding of human neurobiology including cognitive, motor, sensory and behavioral functions.

## Materials and Methods

### Ethics Statement

The use of autopsied or surgical brain tissues and biofluids is part of an ongoing research (Protocol number 2291) approved by the University of Alberta Human Research Ethics Board (Biomedical) and written informed consents were signed before or at the collection time. The protocols for obtaining post-mortem brain samples comply with all federal and institutional guidelines with special respect for the confidentiality of the donor’s identity; blood was collected from live patients and healthy volunteers under the same protocol.

All animal experiments were performed according to Canadian Council on Animal Care (CCAC) (http://www.ccac.ca/en) and local animal care and use committee guidelines. The experiments involving *RAG1^−/−^* and FIV-infected cats were part of ongoing studies (protocol 449 and 452) approved by the University of Alberta Animal Care and Use Committee. The macaque tissues used in this study were obtained from studies approved by the Health Canada Animal Care Committee (ACC Protocols #94084, 96048 and 98033).

### Animal Housing and Care

The brains of cynomolgus macaques were derived from a study on the efficacy of the attenuated SIV vaccine approach in cynomolgus macaques (groups of 4 macaques per arm, including naive control groups). The original macaques were caught in the Philippines under the supervision of a Health Canada Veterinarian and transported to Canada. The colony was free of herpes B virus at the time of tissue collection. Following infection the animals were housed singly in cages in the BSL2/3 housing facility in the Sir Frederick G Banting Research Centre, Health Canada, Ottawa, Canada. Any invasive procedures, such as blood collection were performed under sedation (Ketamine Hydrochloride: 10 mg/kg body weight, IM injection into upper thigh). Euthanization was performed under the supervision of an attending veterinarian and tissues were immediately frozen in liquid nitrogen.

*RAG1^−/−^* mice and FIV-infected cats were housed under specific pathogen free (SPF) conditions in the University of Alberta Health Sciences Laboratory Animals Services facility. For the FIV-infected animals, adult pregnant cats (queens) from an established breeding colony in the University of Alberta Animal Care Facility (founded with cats from U C Davis) were housed and maintained in SPF rooms with appropriate environmental enrichment according to CCAC. Breeding pairs were negative for feline retroviruses (FIV, Feline Leukemia Virus; FeLV) by PCR analysis and serologic testing. Animals were injected intracranially (right frontal lobe) one day post-partum with 200 µl of virus (10^4^ TCID_50_/ml) [44]. Animals were weaned after 6 weeks and monitored over 12 weeks post-infection during which time body weight was measured, neurobehavioral tests performed and blood samples collected. Animals were euthanized by pentobarbital overdose at 12 weeks post-infection and tissues were immediately harvested frozen at −80°C. *RAG1^−/−^* mice were group housed with sex-matched littermates at a maximum of five animals per cage in CCAC-approved cages with daily monitoring by facility technicians and bedding changes bi-weekly. Animals were sacrificed by CO_2_ asphyxiation followed by exsanguination and brain tissue was collected and frozen immediately with N_2(l)_ for later processing.

### Tissue and RNA Collection

Brain tissues were collected at autopsy with consent from HIV-infected (HIV) persons, who were AIDS-defined; HIV-uninfected other disease controls (ODC) as well as from HIV uninfected individuals undergoing surgery for resection of an epileptic focus (SURG) (Table 1). Tissues were stored at −80°C at the Laboratory for Neurological Infection and Immunity Brain Bank at University of Alberta, as previously reported [45], [46]. 0.08–0.2 g cerebral white matter was aseptically excised from autopsy- or surgically-derived brain tissue with sterile instruments, taking care to avoid any visible pathologies including abscesses [24], infarctions or hemorrhages. Brain specimens were placed in Lysing Matrix tubes (MP Biomedicals, Santa Ana, CA, USA) to which 0.8 mL Trizol reagent (Invitrogen, Carlsbad, CA, USA) was added and homogenized in a FastPrep-24 tissue homogenizer (MP Biomedicals, Santa Ana, CA, USA). 0.2 mL of chloroform: isoamyl alcohol (24∶1) was added and the samples were mixed by inversion. The samples were centrifuged 1200 g for 15 minutes at 4°C. The aqueous phase was collected. 1 volume of 70% ethanol was added and total RNA isolated using the miRNeasy kit (Qiagen, Germantown, MD, USA) according to the manufacturer’s protocol. In addition, cerebral tissues were harvested from feline immunodeficiency virus-infected cats (12 weeks old) [44] and *RAG1^−/−^* mice (>52 weeks of age) [47], housed under SPF conditions, from which RNA was prepared as described above. Brain tissues from simian (SIV; n = 10) [48] or simian-human immunodeficiency virus-infected (SHIV; n = 3) [49] or FIV-challenged adult cynomolgus macaques (n = 2) [50] were analyzed by initial preparation of RNA followed by RT-PCR amplification of cDNA and sequencing.

Human brain homogenates (5 µl) with or without prior heat treatment (25 minutes at 65°C) were implanted in the right striatum of 1 year old *RAG1^−/−^* male mice, as previously reported [51], and followed for 7 weeks after which they were sacrificed and brains were harvested for RT-PCR and sequencing.

Blood was collected with consent from healthy volunteers (Protocol 2291). Whole blood was diluted 2× in RPMI (Gibco, Grand Island, NY, USA) and layered over 20 mL histopaque. The samples were centrifuged at 1800 rpm for 22 minutes in a swinging bucket centrifuge. The resulting serum and mononuclear cell layers were collected separately. To extract RNA from serum 0.75 mL of Trizol LS reagent (Invitrogen, Carlsbad, CA, USA), 1 µL of 20 mg/ml glycogen and 0.15 ml chloroform was added to 0.25 mL serum. The samples were manually shaken for 1 minute and left to rest on ice for 3 minutes before being centrifuged at 1200 g for 20 minutes at 4°C. The aqueous phase was collected and 1 volume of 70% ethanol was added. The RNA was then collected with the miRNeasy (Qiagen, Germantown, MD, USA) kit as per the manufacturer’s protocol. The mononuclear cell layer was washed twice in RPMI then re-suspended in 0.8 mL Trizol reagent (Invitrogen, Carlsbad, CA, USA) and total RNA was collected as above.

### Analysis of Deep Sequencing Data

Total RNA from each patient underwent human rRNA subtraction and was then used to construct cDNA libraries and subjected to massively parallel sequencing, as described by Moore *et al.* [52]. Multiplexed reads were deconvoluted according to sequence tags. Reads were aligned sequentially to a human rRNA database, then to the human transcriptome and genome [52]. Matching reads were removed and the remaining reads were aligned to a database of viral and bacterial sequences obtained from Genbank and the Human Microbiome Project. Following this analysis, matching reads were retained if they were above threshold for sequence quality (Q20), did not contain homopolymers, satisfied read pairing logic, and mapped unambiguously (i.e., to only one accession). Finally, putative hits were then subjected to contaminant screening, and were removed if there were any matches to other unrelated RNA-seq libraries generated in house from species other than human. All data were normalized within each run by total tag count. To avoid bias due to inter-run variation, values were first normalized by read length and then a total intensity normalization approach was performed. This approach relied on the assumption that the gene expression profiles between individual samples are similar. The average number of tags for each gene within a sampling run was plotted against the average of the same gene in the sampling run being compared. Linear regression was performed and the slope of the resulting trend line was used to scale the values for comparison between runs. Gene ontology analysis was performed using DAVID Bioinformatic resources 6.7 Functional Annotation tool (http://david.abcc.ncifcrf.gov/). Gene network analysis was performed using Ingenuity pathway analysis (Ingenuity systems, www.ingenuity.com). As the mean bacterial sequence tag counts in brain did not differ between the HIV and ODC groups (and RT-PCR amplification of bacterial 16 s rRNA from brain did not show differences between HIV and ODC groups), gene network pathway analyses of host gene tags were performed using data from all deep-sequenced autopsied brain specimens (n = 8). These calculations showed that total bacterial tag counts per patient (Table 2) were negatively (*r*<−0.7) or positively (*r>*0.7) correlated [44] with host gene tag counts for a subset of host genes (n = 154) among the host genes consistently detected (n = 7038) for all brain specimens. This analysis was extended and verified by calculating the correlation between total bacterial tag counts per patient with those identified 154 host genes, yielding 75 host genes that were significantly correlated with bacterial quantity (Spearman, *p*<0.05) (Table S1).

### Immunohistochemistry

Fixed paraffin embedded brain sections were deparaffinized by 1 h incubation at 60°C, followed by rehydration in xylene through decreasing concentrations of ethanol to water. For antigen retrieval, the samples were brought to a boil in sodium citrate buffer (1 mM, pH 6) then cooled to room temperature. The slides were then blocked with 0.3% H_2_O_2_ for 20 minutes at room temperature, followed by blocking for 2 hrs in Odyssey blocking buffer (LiCor, Lincoln, NE, USA). To detect the bacterial product, peptidoglycan, slides were incubated with a monoclonal anti-peptidoglycan antibody (MAB995 Chemicon, Temecula, CA, USA) (1∶200), or an equal volume of Odyssey blocking buffer as a no primary antibody negative control. Astrocytes were detected with an anti-GFAP (Dako, Carpenteria, CA, USA) (1∶500) antibody. Myeloid cells, including microglia were detected with a monoclonal anti-CD45 antibody (Invitrogen, Carlsbad, CA, USA) (1∶500). The relevant antibodies were applied to each slide and incubated overnight at 4°C. The slides were then washed 3× for 10 minutes in PBS to remove unbound antibody. Biotinylated anti-mouse IgG (1∶500) or anti-rabbit Ig (both supplied by Vector Laboratories, Burligame, CA, USA, Burlingame, CA, USA) (1∶500) antibodies were applied to the slides and incubated 2 hours at room temperature. The slides were again washed 3 × in PBS and incubated with Vectastain Avidin biotin Complex (Vector Laboratories, Burligame, CA, USA) for 2 hours, washed 3× in PBS, then DAB (diaminobenzidine) substrate (Vector Laboratories, Burligame, CA, USA) was applied for 10 minutes; the reaction was stopped by 10 minute incubation in ddH_2_O. The slides were then dehydrated through ethanol to Xylene and mounted with Vectamount (Vector Laboratories, Burligame, CA, USA). For double immunolabeling slides were blocked with levamisole for 20 minutes (Vector Laboratories, Burligame, CA, USA) following DAB staining, then Odyssey blocking buffer for 2 hours (LiCor, Lincoln, NE, USA) then incubated with primary antibody at 4°C overnight. Following three ten minute washes in PBS, alkaline phosphatase conjugated secondary antibodies against the primary anti-IbA-1 (Wako, Richmond, VA, USA) or anti-GFAP (Dako, Carpenteria, CA, USA) antibodies were applied to the slides. Unbound secondary antibody was removed by three, ten minute PBS washes. Finally Alkaline Phosphatase (BCIP/NBT) substrate (Vector Laboratories, Burligame, CA, USA) was applied. The slides were then dehydrated and mounted as described above.

### *In Situ* Hybridization

Slides were deparaffinized and rehydrated as described above. Slides were then treated with 100 µg/mL proteinase K in buffer (50 mM Tris HCl, 10 mM EDTA and 10 mM NaCl) for 10 minutes at 37°C followed by treatment with 10 mg/mL hen egg lysozyme (Sigma, Oakville, ON, Canada) in the same buffer for an additional 20 minutes at 37°C. Slides were then washed twice in PBS and dehydrated through increasing concentrations of ethanol. 150 µL of pre-warmed 2 ng/µL double DIG labeled EUB338 (GCTGCCTCCCGTAGGAGT) probe or scrambled probe in hybridization buffer (25 mM TrisHCl, 100 mM NaCl, 0.5% SDS) was applied to each sampled and incubated at 50°C for 70 minutes. Slides were then washed in rapid changes of 50°C wash buffer (10 mM Tris, pH 9.0, 1 mM EDTA). The samples were then blocked first for 30 min. with levamisole, then for 1 hr with Odyssey blocking buffer (LiCor, Lincoln, NE, USA). A 1∶200 dilution of AP (Alkaline Phosphatase) conjugated sheep anti-DIG Fab’ fragments (Roche, Mannheim, Germany) was applied to the slides and incubated o/n at 4°C. The slides were washed 3 times in PBS and incubated for 2 hours in the dark at 30°C with AP substrate (Roche, Mannheim, Germany), then washed 3 times in PBS, mounted and imaged.

### cDNA Synthesis for PCR and Bacterial RNA Detection and Sequencing

First strand cDNA synthesis was performed using 200 units of Superscript II reverse transcriptase (Invitrogen, Carlsbad, CA, USA) on DNAse (Promega, Madison, WI, USA) treated RNA samples, an equivalent volume of ultrapure water exposed to a hemostat opened but not used in the operating theatre where the surgical specimens were collected, or an equivalent volume of ultrapure water. The resultant cDNA was diluted with 100 µL ultrapure H_2_O for use in subsequent RT-PCR experiments. Multiple host response transcripts were quantified and compared using published primers (Table S2) and cycle conditions [45], [53], relative fold change of each transcript was determined by the delta-delta CT method normalized to the ODC values. RT-PCR detection of bacterial ribosomal RNA (rRNA) was performed using nested PCR protocols to amplify two distinct regions of the 16 s gene. An amplicon from the V8 region of the 16 s gene was generated from brain-derived cDNA using the universal bacterial primers RW01 or RDR080 with DG74 [54]. An initial denaturation step of 3 minutes at 94°C was followed by 35 cycles of 30 seconds(s) at 94°C, 45 s at 59°C and 1 minute at 72°C, followed by a 3 minute final extension at 72°C. Nested PCR reactions were performed using 5 µL of first round amplicon as a template with the primers 16 s1300F and 16 s1450R (Table S3). These reactions were run with an initial denaturation step of 3 minutes at 94°C was followed by 35 cycles of 30 s at 94°C, 45 s at 59°C and 1 minute at 72°C followed by a 3 minute final extension at 72°C. Additional PCR amplifications targeting the V3–V4 region of the 16 s gene were performed. The initial PCR experiment used the primers 16s339F and 16s926R (Table S3). The cycling conditions used for this primer pair were an initial denaturation for 3 minutes at 94°C followed by 20 cycles of 94°C for 30 s, 57.5°C for 45 s and 72°C for 45 s then a final extension step of 3 minutes at 72°C. This amplicon was then used as a template with the primers 16s514F and 16s806R (Table S3) under identical cycling conditions with the exception that an annealing temperature of 56.5°C was used in place of 59°C. All reactions were run alongside a negative control reaction with 5 µL ultrapure H_2_O (Gibco, Grand Island, NY, USA) added in place of template cDNA. Ten µL of each reaction were run on a 1.5% agarose gel impregnated with 4 µg/ml ethidium bromide for approximately 1 hr at 100V in TBE and visualized using an image quant 300 (GE Healthcare, Piscataway, NJ, USA) gel dock. PCR products were excised and extracted using the Qiaquick gel extraction kit (Qiagen, Germantown, MD, USA). 16 s rRNA PCR products were subcloned into pGEM T-easy vector (Promega, Madison, WI, USA) and propagated as per the manufacturer’s instructions in Library efficiency DH5α cells (Invitrogen, Carlsbad, CA, USA). Cells were then harvested and the plasmids were isolated using the GeneJET kit (Fermentas, Burlington, ON, Canada). Purified plasmid was quantified by nanodrop (Thermo Scientific, Waltham, MA, USA) and sequenced in both directions using the T7 and M13R sequencing primers and the Big Dye Terminator 3.1 (ABI, Foster City, CA, USA) Cycle sequencing kit followed by analysis on an ABI 3730 DNA sequencer. Sequences were assembled from bi-directional reads and identified using the BLAST algorithm against the NCBI nucleotide database, in parallel with the RDP (Ribosomal Database project) classifier tool (http://rdp.cme.msu.edu/). Nearest matches were then compared to the brain-derived sequences using the MCoffee alignment suite (http://tcoffee.crg.cat/) or the Clustal X algorithm alone. Phylogenetic trees were constructed using the neighbor joining method with ten thousand bootstrap trials using alignments generated by both methods and including corresponding rRNA sequences from recognized species closely matching the amplified bacterial rRNA sequences. To investigate the presence of bacterially-encoded transcripts, primers targeting *groEL* [55] were used in a semi-quantitative RT-PCR using RT-PCR super mix (BioRAD, Hercules, CA, USA) with autopsy-derived white matter cDNA, surgically-derived white matter cDNA, PBMC-derived cDNA and serum-derived cDNA as templates using an annealing temperature of 56°C. Cycle thresholds were normalized to *gapdh* values from the same run.

### Virus Detection

Detection of herpes viruses was performed by PCR using brain-derived genomic DNA as template with Taqman probes and primers under published conditions (Table S4) [56], [57]. Saffold [58] and Corona [59] viruses were detected in brain-derived cDNA by nested RT-PCR using the primers listed in Table S4.

### Bacterial Transmission

Human cerebral white matter (80 mg) from autopsied ODC13 and 14 brains was homogenized in 800 µL of PBS. The ODC13-derived sample was heat-treated at 65°C for 25 minutes. 2.0 µL of each homogenized human brain suspension was stereotactically implanted into the striatum of *RAG1^−/−^* mice (n = 3) under anesthesia. Animals were maintained under group housing for 7 weeks then sacrificed and the brain collected from which RNA was extracted, as described above for the human samples.

## Supporting Information

Figure S1 Network analyses of host gene expression in relation to bacterial quantity. The identified 75 host genes associated with bacterial quantity were subjected to network analyses generating a network (IPA score 21) emphasizing genes that participated principally in cell morphology, division and maintenance function, e.g. calreticulin, SLC12A, KLF1, netrin (NTN1). Red shading indicates a positive correlation and green shading indicates negative correlation. Thus, there was an apparent association between host genes implicated in essential brain maintenance and functions with bacterial quantity.
(TIF)

Figure S2 Bacterial detection in ODC and HIV patients with or without confirmed HIV encephalitis. Brain specimens were immunolabeled with anti-peptidoglycan antibody. Peptidoglycan-positive bodies were morphologically consistent with bacteria and distributed throughout the tissue (Magnification, left column, 100X) in all patients (Magnification, right column 400X) small clusters of bodies labeled with the anti-peptidoglycan antibody were apparent both perivascularly and in the parenchyma as well as intracellularly (Arrows).
(TIF)

Figure S3 Agarose gel electrophoresis and ethidium bromide staining of 16 s rRNA PCR products. (A) Optimization of primer pairs was conducted by testing compatible primer pairs in a colony PCR protocol using E. coli DH5α as template. All reagents and primer pairs were tested for purity in reaction where ultrapure water was used as a template. Representative gel image testing Reverse primers against a single forward primer is shown. (B) Representative agarose gel depicting amplification products from cDNA made from total RNA extracted from a subset of patients, an equivalent volume of water exposed to a surgical tool opened, but not used, in the surgical suite from which surgical specimens were retrieved, as well as water controls for the first and second round of PCR amplification performed in another facility to confirm the specificity of the finding for the main cohort.
(TIF)

Figure S4 Detection and identification of bacterial 16 s V8 rRNA in human brain from HIV and ODC groups. (A) 16 s V8 rRNA sequences detected in HIV patients relative to ODC patients’ brain specimens by real time RT-PCR and normalized to GAPDH (Mean +/− SD). (B) Phylogenetic analysis of 16 s rRNA V8 region sequences derived from all brain specimens. Clustal alignments were generated comparing amplicon sequences with the equivalent position of published 16 s rRNA sequences identified by BLAST analysis. The Neighbor joining tree was generated based on 10,000 bootstrap trials and rooted on the human mitochondrial 16 s rRNA sequence from the equivalent region.
(PDF)

Table S1 Host genes correlated with bacterial sequence tag abundance.
(DOCX)

Table S2 Primers used for semi-quantitative RT-PCR analysis of host transcript expression.
(DOCX)

Table S3 Primers pairs used to amplify 16 s rRNA.
(DOCX)

Table S4 Primers used to detect RNA or DNA viruses.
(DOCX)
