# Supplementary figures and images for: Brain Microbial Populations in HIV/AIDS: α-Proteobacteria Predominate Independent of Host Immune Status
Source: PLoS One. 2013 Jan 23;8(1):e54673. doi: 10.1371/journal.pone.0054673 (PMC3552853; doi:10.1371/journal.pone.0054673)

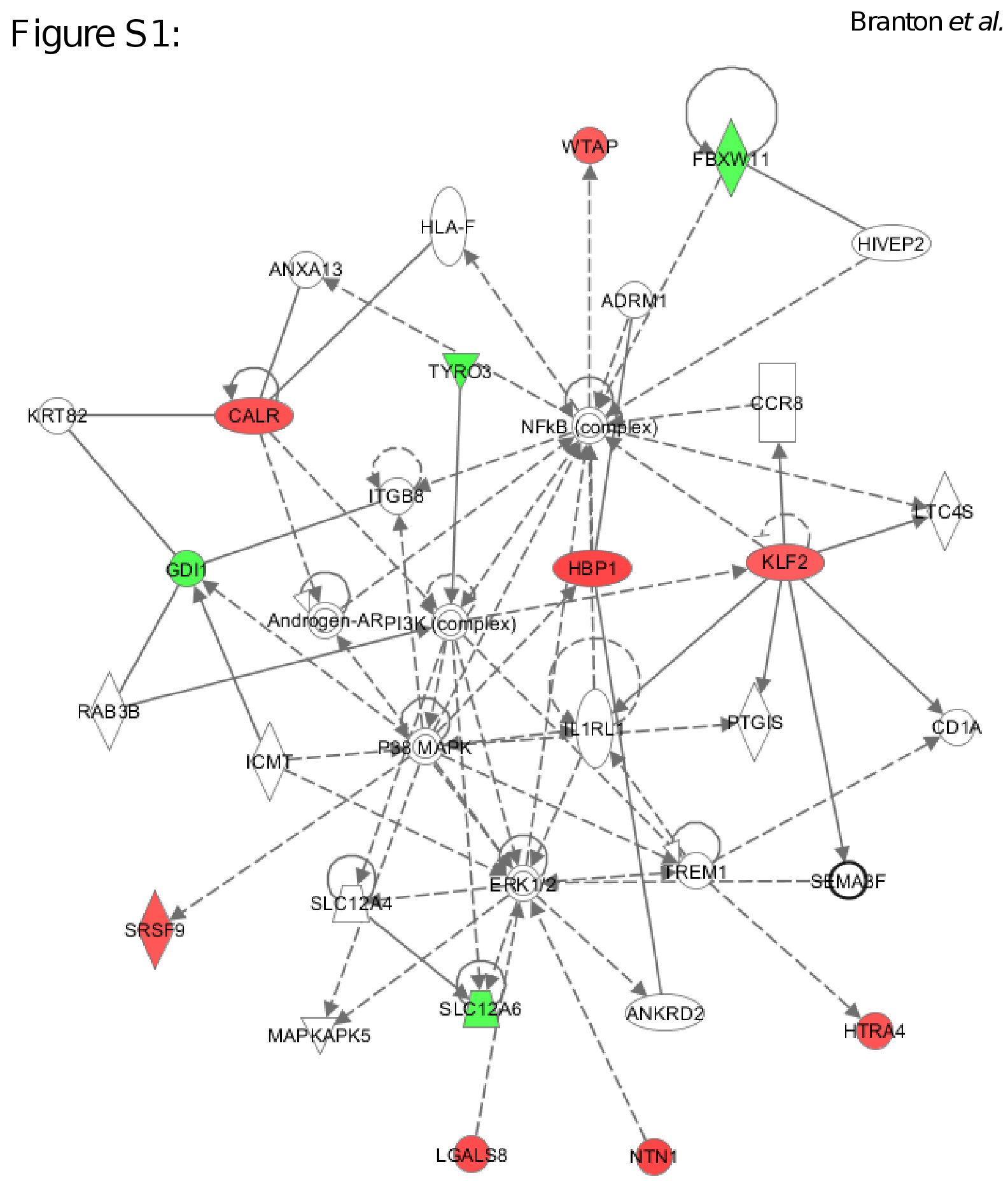

Supplement: Figure S1 — Network analyses of host gene expression in relation to bacterial quantity. The identified 75 host genes associated with bacterial quantity were subjected to network analyses generating a network (IPA score 21) emphasizing genes that participated principally in cell morphology, division and maintenance function, e.g. calreticulin, SLC12A, KLF1, netrin (NTN1). Red shading indicates a positive correlation and green shading indicates negative correlation. Thus, there was an apparent association between host genes implicated in essential brain maintenance and functions with bacterial quantity. (TIF) [file pone.0054673.s001.tif]

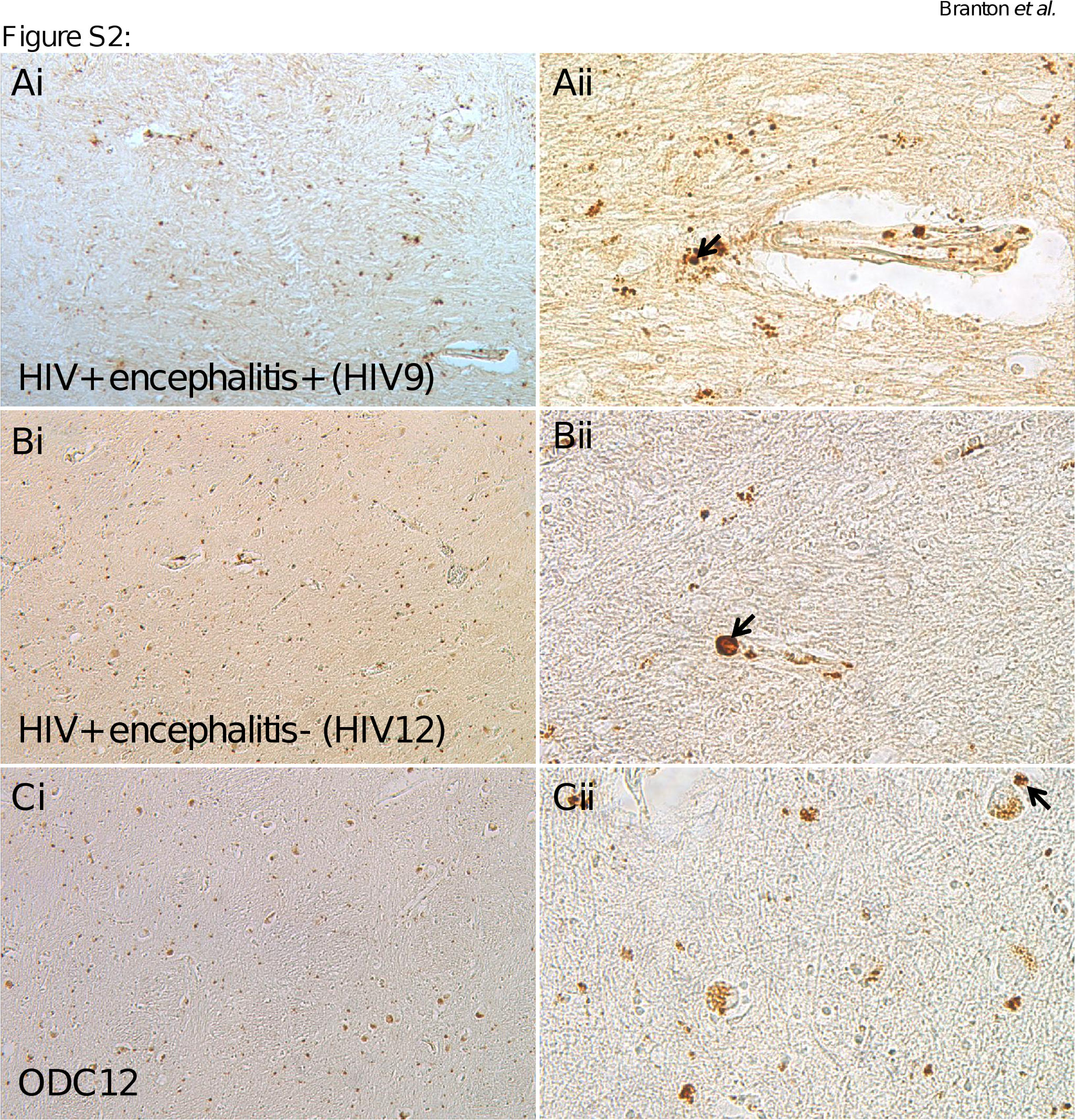

Supplement: Figure S2 — Bacterial detection in ODC and HIV patients with or without confirmed HIV encephalitis. Brain specimens were immunolabeled with anti-peptidoglycan antibody. Peptidoglycan-positive bodies were morphologically consistent with bacteria and distributed throughout the tissue (Magnification, left column, 100X) in all patients (Magnification, right column 400X) small clusters of bodies labeled with the anti-peptidoglycan antibody were apparent both perivascularly and in the parenchyma as well as intracellularly (Arrows). (TIF) [file pone.0054673.s002.tif]

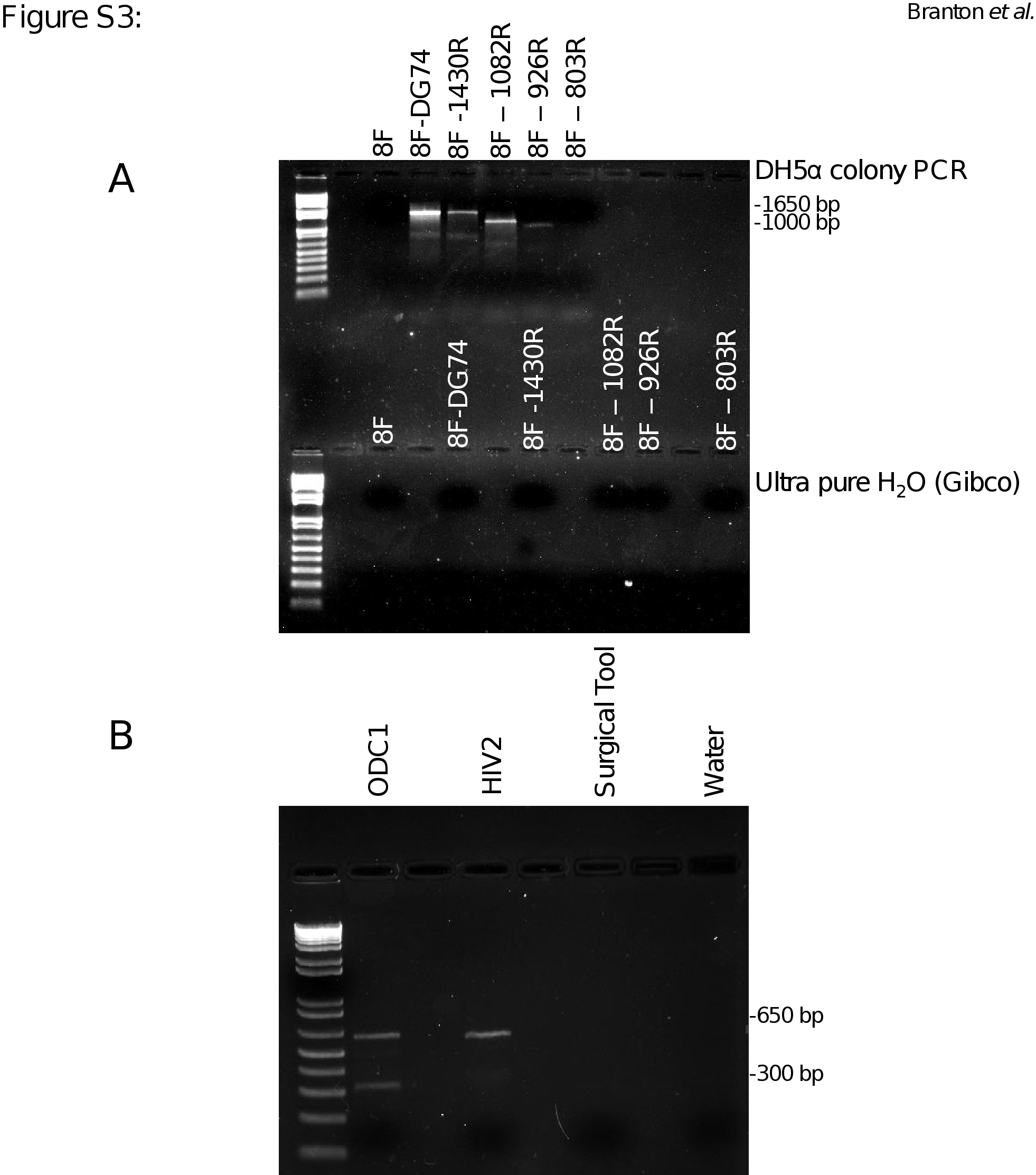

Supplement: Figure S3 — Agarose gel electrophoresis and ethidium bromide staining of 16 s rRNA PCR products. (A) Optimization of primer pairs was conducted by testing compatible primer pairs in a colony PCR protocol using E. coli DH5α as template. All reagents and primer pairs were tested for purity in reaction where ultrapure water was used as a template. Representative gel image testing Reverse primers against a single forward primer is shown. (B) Representative agarose gel depicting amplification products from cDNA made from total RNA extracted from a subset of patients, an equivalent volume of water exposed to a surgical tool opened, but not used, in the surgical suite from which surgical specimens were retrieved, as well as water controls for the first and second round of PCR amplification performed in another facility to confirm the specificity of the finding for the main cohort. (TIF) [file pone.0054673.s003.tif]
