# Supplementary material for: Brain Microbial Populations in HIV/AIDS: α-Proteobacteria Predominate Independent of Host Immune Status
Source: PLoS One. 2013 Jan 23;8(1):e54673. doi: 10.1371/journal.pone.0054673 (PMC3552853; doi:10.1371/journal.pone.0054673)

Figure S4:

Branton *et al.*

A

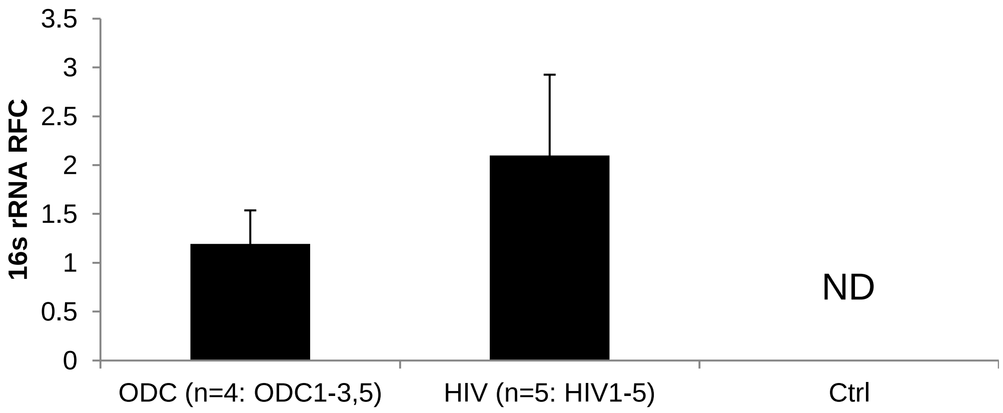

B

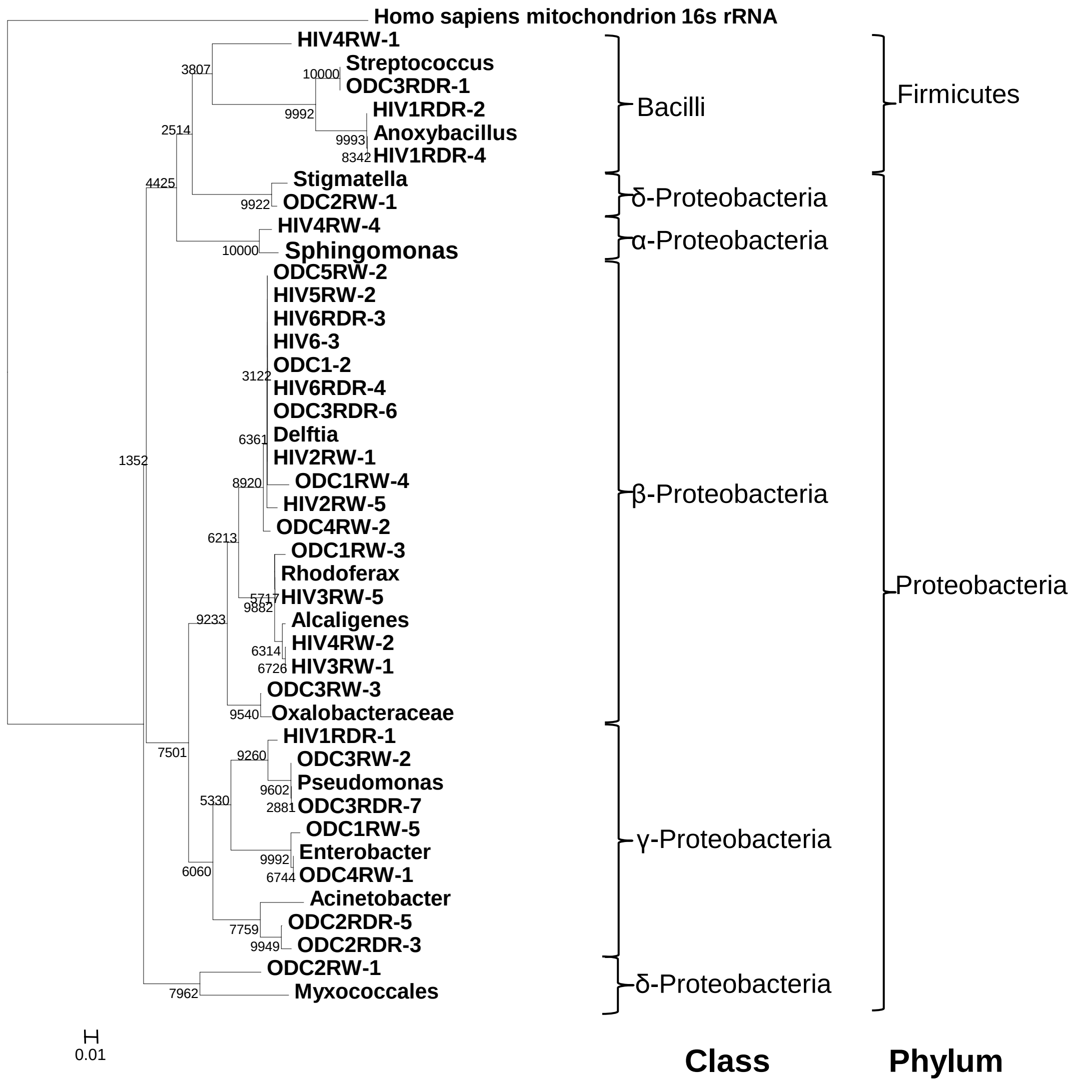

Supplement: Figure S4 — Detection and identification of bacterial 16 s V8 rRNA in human brain from HIV and ODC groups. (A) 16 s V8 rRNA sequences detected in HIV patients relative to ODC patients’ brain specimens by real time RT-PCR and normalized to GAPDH (Mean +/− SD). (B) Phylogenetic analysis of 16 s rRNA V8 region sequences derived from all brain specimens. Clustal alignments were generated comparing amplicon sequences with the equivalent position of published 16 s rRNA sequences identified by BLAST analysis. The Neighbor joining tree was generated based on 10,000 bootstrap trials and rooted on the human mitochondrial 16 s rRNA sequence from the equivalent region. (PDF) [file pone.0054673.s004.pdf]
