# Supplementary material for: Brain Microbial Populations in HIV/AIDS: α-Proteobacteria Predominate Independent of Host Immune Status
Source: PLoS One. 2013 Jan 23;8(1):e54673. doi: 10.1371/journal.pone.0054673 (PMC3552853; doi:10.1371/journal.pone.0054673)
Supplement: Table S1 — Host genes correlated with bacterial sequence tag abundance. (DOCX) [file pone.0054673.s005.docx]

Table S1: Host genes correlated with bacterial sequence tag abundance

| **Transcript** | **r value** | **Transcript** | **r value** | |
| --- | --- | --- | --- | --- |
| coiled-coil domain containing 84 | -0.970 | zinc finger protein 576 | 0.778 | |
| malonyl-CoA decarboxylase | -0.962 | AC096579.14 | 0.779 | |
| carbohydrate (N-acetylgalactosamine 4-0) sulfotransferase 9 | -0.884 | phosphodiesterase 4D interacting protein | 0.780 | |
| KIN antigenic determinant of recA protein | -0.882 | nucleoporin 88kDa | 0.782 | |
| neuroblastoma breakpoint family member 20 | -0.874 | BX571672.2 | 0.785 | |
| TSPY-like 6 | -0.869 | lectin galactoside-binding soluble 8 | 0.800 | |
| Uncharacterized protein C4orf34 | -0.868 | mitochondrial ribosomal protein S5 | 0.808 | |
| zinc finger family member 767 | -0.856 | netrin 1 | 0.829 | |
| zinc finger protein 607 | -0.842 | HMG-box transcription factor 1 | 0.832 | |
| nephronophthisis 3 (adolescent) | -0.837 | C1q and tumor necrosis factor related protein 6 | 0.832 | |
| zinc finger protein 862 | -0.834 | poly(A) polymerase gamma | 0.834 | |
| O-sialoglycoprotein endopeptidase-like 1 | -0.827 | tight junction associated protein 1 (peripheral) | 0.840 | |
| EF-hand domain (C-terminal) containing 1 | -0.826 | serine peptidase inhibitor-like with Kunitz and WAP domains 1 (eppin) | 0.875 | |
| zinc finger protein 132 | -0.821 | Protein SMG9 (Protein smg-9 homolog) | 0.914 | |
| MICAL-like 2 | -0.817 |  |  |  |
| zinc finger protein 845 | -0.817 |  |  |  |
| RP11-261C10.3 | -0.813 |  |  |  |
| Uncharacterized protein C1orf190 | -0.813 |  |  |  |
| RP11-236F9.4 | -0.809 |  |  |  |
| Uncharacterized protein C3orf62 | -0.806 |  |  |  |
| Plasma glutamate carboxypeptidase Precursor | -0.805 |  |  |  |
| septin 7 pseudogene 2 | -0.804 |  |  |  |
| RP11-284F21.5 | -0.802 |  |  |  |
| WAS protein family homolog 6 pseudogene | -0.797 |  |  |  |
| UBX domain protein 4 | -0.791 |  |  |  |
| zinc finger protein 506 | -0.790 |  |  |  |
| TYRO3 protein tyrosine kinase | -0.786 |  |  |  |
| transmembrane protein 170A | -0.784 |  |  |  |
| basic leucine zipper nuclear factor 1 | -0.781 |  |  |  |
| NADH dehydrogenase (ubiquinone) 1 alpha subcomplex 1 7.5kDa | -0.780 |  |  |  |
| RAS and EF-hand domain containing | -0.778 |  |  |  |
| amyloid beta (A4) precursor protein-binding family A member 2 | -0.777 |  |  |  |
| SON DNA binding protein | -0.774 |  |  |  |
| zinc finger protein 397 | -0.774 |  |  |  |
| UPF0550 protein C7orf28 | -0.774 |  |  |  |
| potassium intermediate/small conductance calcium-activated channel subfamily N member 3 | -0.768 |  |  |  |
| G patch domain containing 4 | -0.767 |  |  |  |
| dymeclin | -0.767 |  |  |  |
| RP11-287A8.2 | -0.766 |  |  |  |
| DDRGK domain containing 1 | -0.764 |  |  |  |
| sideroflexin 3 | -0.763 |  |  |  |
| ubiquinol-cytochrome c reductase complex III subunit XI | -0.762 |  |  |  |
| Rab acceptor 1 (prenylated) | -0.758 |  |  |  |
| paired immunoglobin-like type 2 receptor beta | -0.757 |  |  |  |
| GDP dissociation inhibitor 1 | -0.757 |  |  |  |
| COBW domain containing 5 | -0.755 |  |  |  |
| ubiquitin-conjugating enzyme E2Z | -0.754 |  |  |  |
| ArfGAP with coiled-coil ankyrin repeat and PH domains 3 | -0.753 |  |  |  |
| dynein cytoplasmic 1 light intermediate chain 1 | -0.748 |  |  |  |
| coiled-coil domain containing 66 | -0.747 |  |  |  |
| DEAH (Asp-Glu-Ala-His) box polypeptide 37 | -0.747 |  |  |  |
| zinc finger protein 800 | -0.746 |  |  |  |
| pecanex-like 3 | -0.745 |  |  |  |
| membrane-associated ring finger | -0.744 |  |  |  |
| protocadherin gamma subfamily C 3 | -0.743 |  |  |  |
| Probable E3 ubiquitin-protein ligase KIAA0614 | -0.742 |  |  |  |
| zinc finger and BTB domain containing 20 | -0.742 |  |  |  |
| breast carcinoma amplified sequence 3 | -0.740 |  |  |  |
| RAB member of RAS oncogene family-like 2A | -0.739 |  |  |  |
| solute carrier family 12 (potassium/chloride transporters) member 6 | -0.736 |  |  |  |
| arginine-glutamic acid dipeptide (RE) repeats | -0.736 |  |  |  |
