# Supplementary material for: Brain Microbial Populations in HIV/AIDS: α-Proteobacteria Predominate Independent of Host Immune Status
Source: PLoS One. 2013 Jan 23;8(1):e54673. doi: 10.1371/journal.pone.0054673 (PMC3552853; doi:10.1371/journal.pone.0054673)
Supplement: Table S2 — Primers used for semi-quantitative RT-PCR analysis of host transcript expression. (DOCX) [file pone.0054673.s006.docx]

Table S2: Primers used for semi-quantitative RT-PCR analysis of host transcript expression.

| **Primer** | **Sequence** |
| --- | --- |
| Hu HLA-DRA fwd | GGACAAAGCCAACCTGGAAA |
| Hu HLA-DRA rev | AGGACGTTGGGCTCTCTCAG |
| hu_IL-10-fwd-1 | AATAAGGTTTCTCAAGGGGCT |
| hu_IL-10-rev-1 | AGAACCAAGACCCAGACATCAA |
| hu_IL-4-fwd 1 | AGAAGACTCTGTGCACCGAGTTGA |
| hu_IL-4-rev 1 | CTCTCATGATCGTCTTTAGCCTTT |
| hu_IL-6F | ACCCCTGACCCAACCACAAAT |
| hu_IL-6R | AGCTGCGCAGAATGAGATGAG |
| hu IL-23p19 fwd 1 | GAGCCTTCTCTGCTCCCTGAT |
| hu IL-23p19 rev 1 | AGTTGGCTGAGGCCCAGTAG |
| Hu CD3e fwd | GATGCAGTCGGGCACTCACT |
| Hu CD3e rev | CAT ACCATCTTGCCCCCAA |
| hu IL-12p35 fwd 1 | AGCCTCCTCCTTGTCGCTACC |
| hu IL-12p35 rev 1 | GCCTCCACTGTGCTGGTTTTATC |
| Hu PD-L1 (cd274) fwd | CTTCAAGCAGGGATTCTCAACCT |
| Hu PD-L1 (cd274) rev | TAAGTCCCACATTGCCTGCAT |
| mus IFN-alpha fwd | AGGACAGGAAGGATTTTGGA |
| mus IFN-alpha rev | GCTGCTGATGGAGGTCATT |
| mus IL-12p35 fwd 1 | CATCGATGAGCTGATGCAGT |
| mus IL-12p35 rev 1 | CAGATAGCCCATCACCCTGT |
| IL-1b-F | CCAAAGAAGAAGATGGAAAAGC |
| IL-1b-R | GGTGCTGATGTACCAGTTGGG |
