# Supplementary material for: Brain Microbial Populations in HIV/AIDS: α-Proteobacteria Predominate Independent of Host Immune Status
Source: PLoS One. 2013 Jan 23;8(1):e54673. doi: 10.1371/journal.pone.0054673 (PMC3552853; doi:10.1371/journal.pone.0054673)
Supplement: Table S3 — Primers pairs used to amplify 16 s rRNA. (DOCX) [file pone.0054673.s007.docx]

Table S3: Primers pairs used to amplify 16s rRNA.

| **Forward** | **Sequence** | **Reverse** | **Sequence** |
| --- | --- | --- | --- |
| RW01 | AACTGGAGGAAGGTGGGGAT | DG74 | AGGAGGTGATCCAACCGCA |
| RDR080 | AACTGGAGGAAGGTGGGGAC | 16s1450R | TACCTTGTTACGACTTCACCCC |
| 16s1300F | ACACACCGCCCGTCACACC | 16s926R | CCGTCAATTCCTTTRAGTTT |
| 339F | CTCCTACGGGAGGCAGCAGT | 16s806R | TCATCGTTTACGGCGTGGACTACC |
| 16s514F | CGTGCCAGCAGCCGCGGTAAT | 16S803R | CTACCAGGGTATCTAA |
| 16s8F | AGAGTTTGATCCTGGCTCAG | 16s1082R | GGGTTGCGCTCGTTGCGG |
