# Supplementary material for: Brain Microbial Populations in HIV/AIDS: α-Proteobacteria Predominate Independent of Host Immune Status
Source: PLoS One. 2013 Jan 23;8(1):e54673. doi: 10.1371/journal.pone.0054673 (PMC3552853; doi:10.1371/journal.pone.0054673)
Supplement: Table S4 — Primers used to detect RNA or DNA viruses. (DOCX) [file pone.0054673.s008.docx]

Table S4: Primers used to detect RNA or DNA viruses.

| Target Virus | Primer/Probe | Sequence |
| --- | --- | --- |
| HSV | HSV-1 fwd 1 | TTCTCGTTCCTCACTGCCTCCC |
|  | HSV-1 probe 1 | CGTCTGGACCAACCGCCACACAGGT |
|  | HSV-1 rev 1 | GCAGGCACACGTAACGCACGCT |
| VZV | VZV-fwd 1 | CGGCATGGCCCGTCTAT |
|  | VZV-probe 1 | ATTCAGCAATGGAAACACACGACGCC |
|  | VZV-rev 1 | CTCGCGTGCTGCGGC |
| HHV-6 | HHV-6ab fwd 1 | GACAATCACATGCCTGGATAATG |
|  | HHV-6ab rev 1 | TGTAAGCGTGTGGTAATGGACTAA |
|  | HHV-6ab-Probe 1 | FAM-AGCAGCTGGCGAAAAGTGCTGTGC-TAMRA |
| EBV | EBV-fwd-1 | AAACCTCAGGACCTACGCTGC |
|  | EBV-probe 1 | TAGAGGTTTTGCTAGGGAGGAGACGTGTG |
|  | EBV-rev-1 | AGACACCGTCCTCACCAC |
| CMV | CMV fwd 1 | GGCCGTTACTGTCTGCAGGA |
|  | CMV probe 1 | CCGTATTGGTGCGCGATCTGTTCAA |
|  | CMV rev 1 | GGCCTCGTAGTGAAAATTAATGGT |
| HCoV | HCoV229EE7 | TCTGCCAAGAGTCTTGCTCG |
|  | HCoV229EE7.1 | CAAAAGAACAAAAGCARGAAATCG |
|  | HCoVE9 | AGCATAGCAGCTGTTGACGG |
|  | HCoVE9.1 | GCTCAGCAAATTGTGGATAGC |
| SAFV | SAFV-F | CCCCCTTCAATTATAAGATTACACC |
|  | SAFVF2 | GGACGATTGTTCTGACAACT |
|  | SAFVR | AGCTTTTCCTTTAGAGTACCTGG |
|  | SAFVR2 | GCTAACCATTGCTTTCAAAT |
